# Quacks and Quack Medicines

**Published:** 1829-01-01

**Authors:** 


### QUACK MEDICINES.

"They manage these things better in France."

The above epigraph, from Sterne, being in the *present tense*, is applicable; but it would not have been available three months ago. In the eleventh and thirteenth years of the republic, "one and indivisible," the most severe prohibitory laws against quack medicines and secret nostrums were enacted in France, and apothecaries (pharmaciens) corresponding with our chemists and druggists, were strictly forbidden to keep, sell, or dispense, any medicine not contained in the Pharmacopœia, or prescribed by a regular physician or surgeon. These wise laws either fell into total disuse—or probably were never put in force; and, up to the Autumn of 1828, the trade of *quack-medicine-vending* was full as flourishing in Paris as in London. We believe that the French Government, however, derived but little revenue from the sale of patent medicines and their advertisements;—hence probably one cause of the recent revival of the revolutionary edicts against the trade of quackery. The prefect of police has now given a death-blow to the Eadys, the Jordans, the Solomons, &c. of Paris, by prohibiting the sale of their invaluable specifics —and not merely the sale, but even the *mention* of them (by way of advertisement) under the penalty of fine and imprisonment! We hope—or at least we expect, that no such tyrannical laws shall be imported into this country. It would evidently be an infringement on his liberties, as settled by Magna Charta and the glorious revolution, if JOHN BULL were to be prohibited from poisoning himself or his neighbour, in the way of *trade*—and when no *malice prepense* as to murder or suicide, could be proved. When, indeed, we consider that both the vendor and the swallower of a patent medicine are entirely ignorant of the nature of the drug and of the disease for which it is taken, nothing can be more innocent than the whole transaction. In such cases, the *intention* is every thing, and the *event* nothing. Besides, who is unaware that there is a destiny hanging over us all, from which we cannot escape. If a sparrow falls not to the ground without the permission of Providence, neither does a man. In physic, as well as in war, every bolus and every bullet has its billet—and to that billet it will go in spite of all laws and ordonnances.

Our Government takes a more philosophic and *liberal* view of the subject of quackery and quack medicines than the governments of the Continent. It is well aware that there is a redundant population in this country—that redundancy leads to penury—penury to crime—crime to transportation—and transportation to a heavy expense. Now, Mr. Malthus has not pointed out a more efficient check to redundancy of population, than quackery and quack medicines—and we maintain, without fear of contradiction, that this *salutary* check is, of all others, the least shocking to our feelings. It is, in short, a *gilded pill*, by means of which the KING of TERRORS is made to approach in the attractive shape of the Goddess HYGEIA!

It is disagreeable to descend from these sublime and comprehensive views of an important subject in political economy to the mercenary consideration of the effects of quackery and quack medicines on the interests of the medical profession. There is a general opinion afloat that quackery and domestic medicine only make serious work for the regular practitioner. *Serious* it often is! The time for active, and consequently for effectual treatment, in acute diseases, is very frequently absorbed in the application of domestic or patent medicines, and the practitioner is called in when the resources of art are unavailing. It is hardly necessary to remark that the doctor's revenue is derived from the living, and not from the dead. On the other hand, where disorders are so mild that NATURE overcomes both them and the unsuitable treatment by the patent nostrum or family medicine-chest, the regular practitioner is still a loser. But—

"All partial ill is universal good;"

And therefore we cannot but applaud and admire the philanthropy as well as the philosophy of our Government, in fostering and protecting quack and patent medicines, as engines that roll great revenue into the public exchequer, while they disencumber the community of many thousand *unproductive* members annually, by translating them to other and better climes than Australia or Canada.

---

